# Investigating Outcome Types and Betting Strategies in Roulette: A Simulation Study

**DOI:** 10.1159/ear/acrag004

**Published:** 2026-10-15

**Authors:** Matthew King-Parker, Steve Sharman

**Affiliations:** a University of Lincoln, College of Science and Health, School of Psychology, Sport Science and Wellbeing, Lincoln, United Kingdom; b King’s College London, National Addiction Centre, London, United Kingdom; c Loughborough University, School of Sport, Exercise, and Health Sciences, Loughborough, United Kingdom

**Keywords:** Roulette, Gambling, Addiction, Near misses, Loses disguised as wins

## Abstract

**Introduction:**

Roulette has not received as significant attention in research as other forms of gambling. Where roulette has, betting style is often constrained, reducing ecological validity. The present study sought to understand different betting strategies, potential outcome distributions, and implications for research.

**Methods:**

To build an understanding of the frequency of types of outcomes in roulette, the present research used a virtual roulette game to simulate 22,000 spins on a roulette wheel across 11 different betting strategies. There is the potential for 17 different outcome types for roulette: win, loss, loss disguised as a win, and 14 different types of near miss.

**Results:**

Across the 11 different strategies, the rates of the different outcomes varied from being the minority to the majority outcomes. These data showed that roulette outcomes vary significantly as a function of betting strategy and that certain outcomes may not be encountered. However, the amount earned over the strategies did not differ from their starting value.

**Conclusions:**

Experimental research cannot allow participants to gamble organically and simultaneously retain experimental control over outcome distribution. Therefore, for roulette studies, researchers have three potential options: (1) To use pre-determined outcome sequences with restricted betting choices; (2) use a pseudo-randomised sequence with programmed percentages of outcomes that allows ecologically valid betting but using a larger number of trials; (3) use a random sequence and control for outcome variance using via statistical analysis. This study demonstrates factors researchers must consider when balancing experimental control and ecological validity in experimental roulette tasks.

## Introduction

Gambling is common behaviour amongst UK adults. Data from Gambling Commission, the government entity responsible for regulating gambling in the UK, show that 61% of the adult population have gambled in the last 12 months, and 48% have gambled in the last 4 weeks [1]. Further figures indicate that 2.5% of the population experience disordered gambling, with a further 12% demonstrating a low-to-moderate risk of harm [2]. Casino games – including roulette – are gambled on by approximately 4% of gamblers [1].

Much of the experimental research around decision-making in gambling has utilised slot machines (real or simulated), and consequently, roulette as a game has received less empirical focus [3, 4]. This is largely due to the complex betting methods in roulette compared to other games that can result in significant differences in outcomes. This makes it difficult to use rigged outcome sequences and the ability to obtain generalisable results. Scientifically, rigorous and controlled research in other gambling games has led to changes in policy about how casino games are allowed to operate. For example, statutory stake limits for online slot machines and having distinguishable auditory events for wins versus losses disguised as wins (LDWs) [5, 6]. These results can only be obtained through rigged outcomes sequences. As such, without the methodological considerations in designing roulette tasks, rigorous research examining the risks and harms of game mechanics cannot be conducted.

To research the game in an ecologically valid manner – and to not artificially over-restrict betting options – whilst also retaining experimental control has proven difficult. Roulette as a game has two stages. The initial betting stage allows all players to bet on their predicted outcomes across the available bets on the table. The board that players can bet on is ordered numerically (see Fig. 1), whereas the wheel is not ordered numerically. Once all bets have been set, stage 2 begins, and the ball is thrown onto the wheel. A winning number is decided by where the ball settles. Each number can contribute to a “winning spin” in multiple ways, based on its colour and position on the board.

**Fig. 1.**
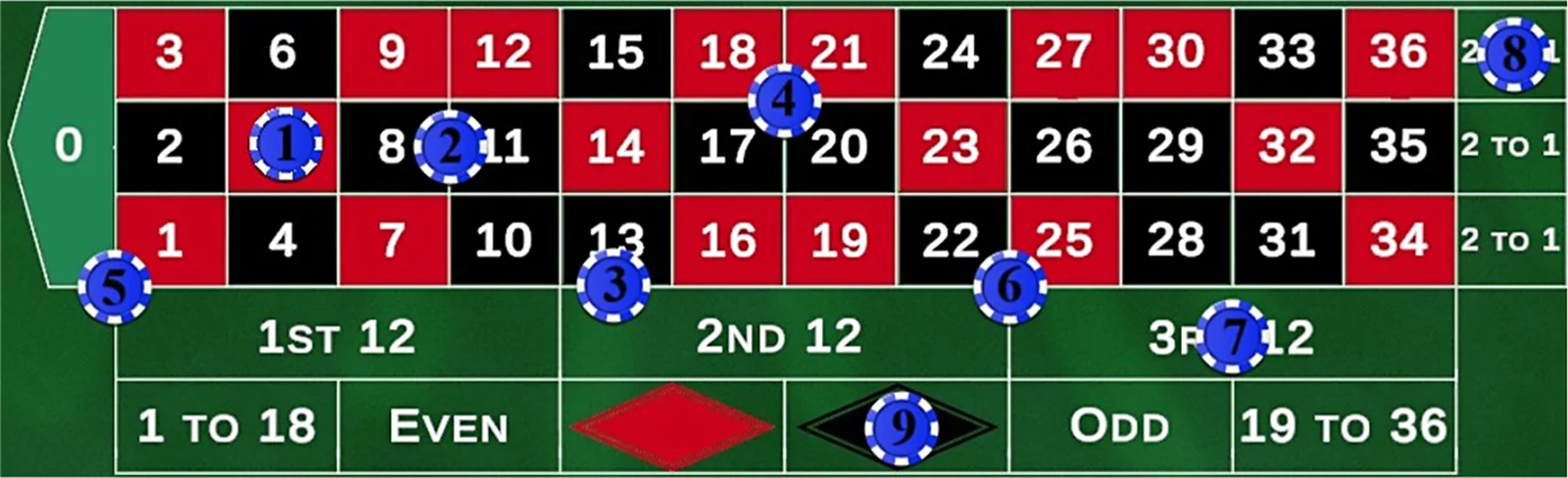
Roulette board showing the major types of bets that can be placed.

In roulette, there are several ways to bet resulting in a variety of potential outcomes based on the betting strategy. There are at least 9 different types of bets that can be placed including (1) straight bet – betting on 0–36, (2) split – placing a bet between two numbers (i.e., 2 and 5), (3) street bet – placed at the bottom of column cover the three spaces, (4) corner bet – a bet placed in the corner between 4 spaces, (5) street bet including 0 (and 00) – covers the column of 1, 2, and 3 as well as including 0 and 00, (6) line bet – placed in the middle of two columns spreading the bet over 6 spaces, (7) dozens – 1st, 2nd, or 3rd 12 numbers, (8) rows – top, middle, and bottom rows of 12 numbers, (9) 50/50 bets – bets on, 1–18, even, red, black, odd, and 19–36 (Fig. 1). Each of these bet types can be placed in combination with other bets to form larger strategies that account for different board coverage and consequently different potential outcomes. As a number of bets can be placed and in combination with each other, there are strategies that can result in comprehensive board coverage, almost guaranteeing some form of win – but with a low win magnitude. Adopting the converse approach, some strategies can be devised that have high win magnitudes but low odds of a win.

For common roulette strategies that can be seen being implemented by players, the vast majority of strategies of betting are based on the amount bet per round. For instance, the Martingale strategy doubles the bet on every loss or the reverse on every win. Similarly, the D'Alembert strategy increases the bet amount by 1 per loss and reduces by 1 per win. There is also the Fibonacci sequence where the player increases the amount bet in line with the sequence (1, 1, 2, 3, 5, 8, 13, 21, 34, 55, 89, 144, etc.) on every loss and decreases each win. Fewer strategies are designed with placement of the bets in mind. There are less recognised strategies where placement of the bet takes precedence over quantity, such as the James Bond strategy. This strategy requires the gambler to put 70% of their pot on 19–36, 25% on a double street bet of 13–18, and the remaining 5% on 0. This strategy covers a large portion of the board and guarantees a high win chance with varying magnitudes without the chance of an LDW. The betting strategies used by a player may potentially have an impact on the prevalence of problematic gambling. A betting strategy with high win magnitudes but lower win frequencies may lead to loss chasing, whereby players increase their betting in an attempt to win [7]. Opposingly, higher win frequencies with a lesser win magnitude may incur a differing psychological impact on the player.

There are rules placed on betting strategies in roulette, whether it is in casinos or electronically. These include a limit on the number of bets per spin, stated maximum and minimum stakes for bets, and limits on the number of spaces that can be bet on in one game. Due to the complexity of this, a number of studies that have utilised a roulette-type task have attempted to limit the number of spaces a participant can bet on, by either limiting to just 50/50 bets, or removing split, street, corner, and/or line bets, meaning gamblers can only bet on the direct number or the 1/3 or ½ bets [3, 4]. This artificially constrains the ability of the gambler to bet in a naturalistic way, which may restrict the generalisability of findings. Allowing realistic roulette gambling in an experimental context means that an experimental manipulation cannot have a predetermined outcome sequence as participants may bet in a capacity that does not allow for that outcome (i.e., lose, LDW, or near miss [NM]) to be present. As such, when designing an experimental roulette task, if participants are to be allowed to bet freely and in a naturalistic way, the prevalence of behaviour-impacting outcomes must be considered.

### Near Misses

NMs occur when the gambler comes close to winning but in fact still loses. Examples of an NM can include a horse picked to win coming in a close second, the selected players hitting the post or missing a good chance in a first goal scorer footballer bet, or, as most commonly used in experimental research, two reels matching a win symbol on a 3 reel slot machine, but the third reel settling on a different symbol. NMs are thought to be important in the development and maintenance of gambling disorder and have been shown to encourage persistence gambling [5]. Previous research indicates that NMs elicit a bivalent psychological response of being both more aversive than other non-win outcomes yet simultaneously increasing motivation to continue gambling [6]. Furthermore, the frequency of NMs is known to increase the length of play within games [7]; previous research has suggested that NMs drive increased gambling (as operationalised by increased persistence) when they occur on 30% of spins when compared to 15 and 45% of spins. When occurring on only 15% of spins, the NM is not consistently predictive of a win, therefore lack reinforcement properties; when occurring on 45% of spins, the NM as the secondary reinforcer is not paired consistently enough with the primary reinforcer (a win) and therefore reinforcing properties are nullified. Therefore, NMs on 30% of spins (trials) drive increased persistence. NMs have also been shown to increase subsequent bet size on the next spin (compared to full misses) [8, 9]. Despite the fact that they are objectively identical to “full” loses, several studies have shown a difference in psychological and physiological response to NMs, with NMs eliciting neuropsychological and psychophysiological responses that are more closely aligned to a win profile, than a loss profile [5, 10–13].

It should be noted that experimental support for the NM effect is not universal; a recent review identified several methodological issues with oft-cited studies including lack of direct behavioural measures of gambling, outcome sequences, number of trials, and replication failure [14]. When addressing said methodological limitations, both animal and human studies failed to replicate the NM effect. However, subsequent work indicates that Pisklak and colleagues [15] may themselves have designed an experimental paradigm that neglected specific boundary conditions such as visual cues (e.g., 3 symbols vs. a more commonly observed multi-line view) and visual dynamics (e.g., stationary stimuli vs. spinning reels, sequential stopping) [9].

### NMs in Roulette

Although within a specific game the return to player may be fixed, the proportional distribution of outcomes across multiple spins (e.g., true wins, NMs, and LDWs) is not and may differ based on an individual’s betting strategy. However, few studies have investigated the potential role of NMs in roulette [3, 16]. Sundali et al. [16] highlight that there may be two types of NMs in roulette: board and wheel NMs. A board NM occurs when the placed bet is next to the winning space (i.e., bet on 5 and the winning number is 6), whilst a wheel NM focuses on where the ball lands on the wheel, whether that is next to the number on which the bet has been placed on the board (e.g., 5 on the board and 10 on the wheel). Sundali et al. [16] found that near misses did not lead players to gamble for longer. Instead, the near miss affected where the next bet was placed. For some of the sample, it was found they placed bets away from the near miss, in a gambler’s fallacy type bias, whilst others bet on the near miss number, now believing the likelihood of a win higher. Although providing some important context for NM roulette studies, this study was a field study, therefore lacks experimental control, and thus should be interpreted with caution. Dixon [3] found that participants rated losses closer to wins based on the number value of the win being close to the bet number and the proximity on the roulette table. However, this paper only attributes board NMs and does not discuss the potential for wheel NMs.

### Losses Disguised as Wins

LDWs are a particular type of outcome that depending on betting strategy and can be very common in roulette. An LDW occurs when the gambler wins an amount that is less than the total amount staked. So, although the spin/bet returns something, the amount is less than staked; therefore, it is objectively an overall loss. The psychological importance of LDWs in persistent gambling is thought to be associated with two important constructs in slot machine design – payback percentage and reinforcement rate.

On a slot machine, gamblers can often choose how many “lines” are activated with each spin and pay per line, for example, if, on a machine where a single line costs 25 pence, the gambler can select a single line where each spin will cost 25 pence and will offer one opportunity to win. However, if the gambler chooses 10 lines, each spin will cost GBP 2.50 but will offer 10 different potential win lines.

Harrigan and colleagues [17] used the slot machine game “Moneystorm” to simulate 5,000 spins betting on a single win line and 5,000 spins betting on 20 win lines. When betting on one line, the simulation produced a win percentage of 15.4%, resulting in a reinforcement rate of once every 6.5 spins. When the number of lines bet on was increased to 20 per spin, the win percentage was 47.9, and the reinforcement rate increased to every 2.1 spins. The total credits won on the single line game was 4,525, compared to 90,504 on the 20-line game; however, the total amount wagered on the single line game was 5,000 credits, compared to 100,000 in the 20-line game. This results in an identical payout rate of 90.5%, highlighting how LDW increases reinforcement rate without changing the payback percentage. This can change the perception of a gambling session and highlights importance of controlling this type of outcome in an experimental roulette task.

### Present Study

The aim of the present study was to explore how to design a rigorous and ecologically valid experimental research task for roulette gambling. Due to the inherent variability in gambling strategies and the importance of each event type in influencing gambling behaviour, the present study sought to examine the distribution of outcome types across different roulette betting strategies. The present study aims to examine the outcome ratios of simulated roulette games as a function of different strategies. Exploratory analysis examined whether:

- outcome proportions (e.g., win, NM, LDW) vary as a function of roulette strategy;
- different roulette strategies result in variability in overall strategy winnings.

## Methods

### Roulette Simulation

A roulette game was developed in Unity 2022.3.0f1. The roulette game works in a similar way to a real-world European/single-zero roulette wheel (see Fig. 1, 2). The logic used to dictate the roulette wheel’s outcomes was set to randomly select 1 of the 37 numbers on the roulette wheel each new game; this included previous winning numbers.

**Fig. 2.**
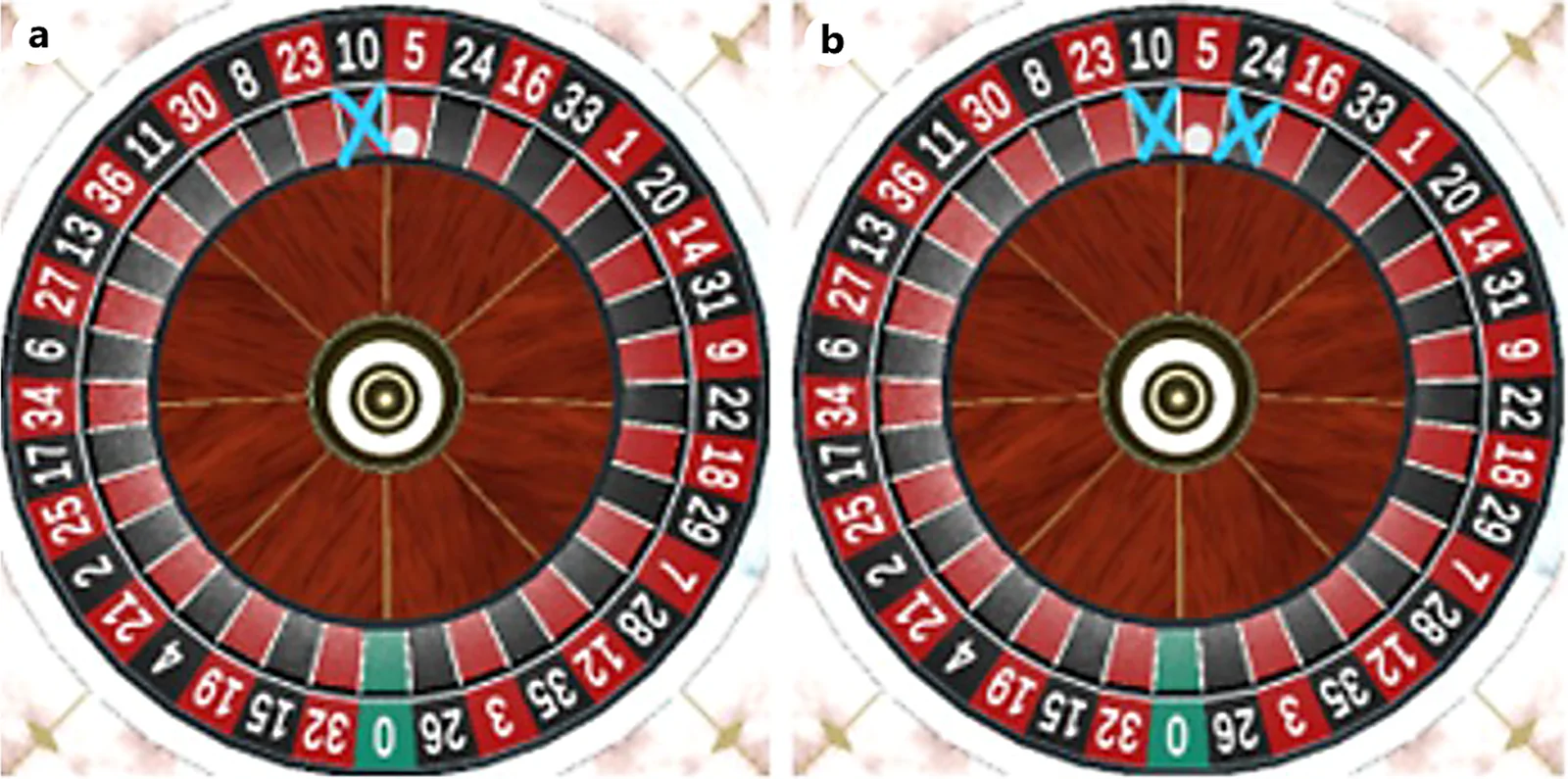
The diagrams show wheel NMs where the winning number is 5. Blue crosses represent the bet on spaces. **a** Single-wheel NM. **b** Double-wheel NM.

The data for the present research was generated from 22,000 spins of a roulette wheel. Due to the game using a random logic for the outcomes, there was not an even distribution of numbers across the 22,000 games. The aim was to understand the types of outcomes and the frequencies of each type of betting strategy employed.

### Potential Outcomes

There are 5 major outcome types in a game of roulette: loss, LDW, win, wheel NM, and board NM. However, the wheel and board NMs have the potential to compound creating at the minimum a possibility of 14 different NM types: board NM (single, double, triple, and quadruple adjacent board numbers), wheel NM (single and double adjacent wheel numbers), and the combination of the board and wheel NMs (i.e., double wheel NM, with triple board NM). For example, where the winning number is 5, there is the potential for two wheel NMs (see Fig. 2), where the player has bet on 10 or 24. There is also the possibility for four different board NMs, if the player bets on 2, 4, 6, and 8 (see Fig. 3). Each of these can be combined together to form a variety of different outcome combinations of NMs, as well as the potential to be paired with losses, LDWs, and wins. In this study, NMs are only considered for single number bets, not for ½ or 1/3 bets.

**Fig. 3.**
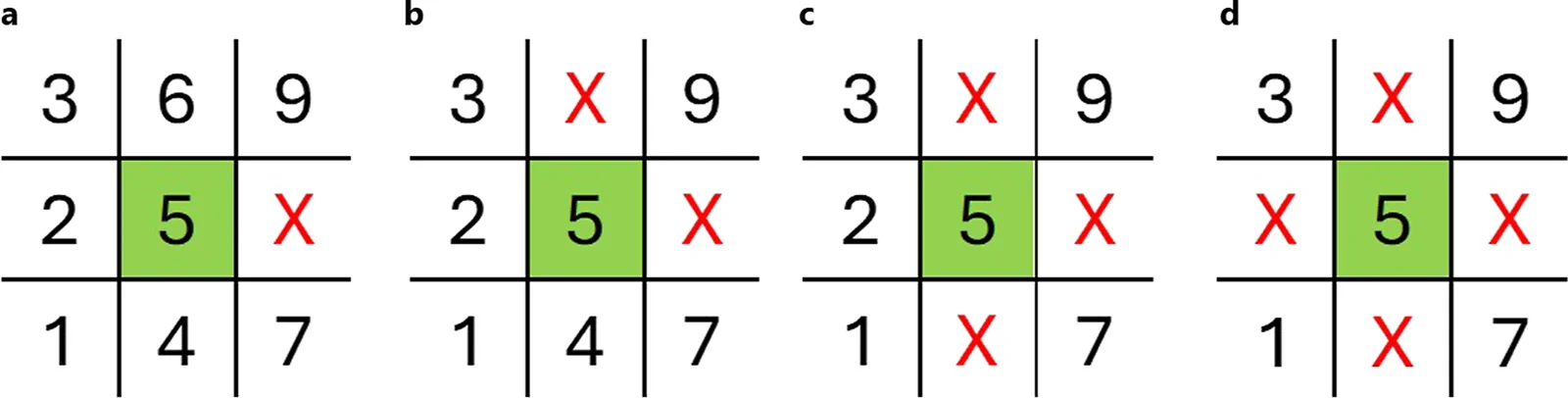
The diagrams show board NMs where the winning number is 5. Red crosses represent the bet on spaces. **a** Single-board NM. **b** Double-board NM. **c** Triple-board NM. **d** Quadruple NM.

### Roulette Strategies

To understand the impact on outcome distribution of betting strategy, 11 different betting strategies were devised. Each strategy was used for 2,000 games of roulette where each spin bet 50 credits across a maximum of 10 board spaces and was loaded with 100,000 credits to ensure that all 2,000 games could be played. The strategies were based on existing popular strategies available on online, were edited in a variety of manners but were made so that they could not guarantee a win no matter the outcome (i.e., betting on red, black, and 0). The strategies only included straight, dozens, rows, and 50/50 bets. The strategies were selected to have a range of low to high win magnitude (strategy 2 vs. 6) bets and a combination where possible (i.e., strategy 3.A). The strategies were also designed to evoke different proportions of outcomes with a range of win chances (see Table 1 for the details for each strategy).

**Table 1.** Roulette strategies used

Strategy	Bets placed ((*credits*) bet on space)	Win percentage	Min.–Max. magnitude	Other potential outcomes	Aim of strategy
1	(*35*) 19–36, (*13*) 2nd 12, and (*2*) No. 0	51%	39–109	There will be no NMs, with a potential for wins, LDWs, and losses	The aim of this strategy was to cover a significant proportion to give a win, similar to the James Bond strategy
2	(*20*) 1st 12, (*20*) 2nd 12, and (*10*) top row	65%	30–90	There is no chance for an NM of any kind, resulting in only losses, LDWs, and wins	This strategy covers a large proportion of the board, leaving only 7 spaces not bet on
3.A	(*35*) even, (*10*) No. 20, and (*5*) No. 8	48%	70–430	There are no chances of an LDW but opportunities for losses, board NMs, wheel NMs, and wins	This covers a significant part of the board with a two lucky-guess bets. Numbers 20 and 8 are being used as lucky numbers, but they are even, so it does not increase the odds
3.B	(*35*) even, (*10*) No. 7, and (*5*) No. 13	54%	70–360	There is a chance for wheel NMs and losses but no other outcomes	This strategy is the same as the prior, but now the lucky numbers are odd, increasing the chances of a win
3.C	(*35*) even, (*10*) No. 7, and (*5*) No. 18	51%	70–360	There are chances of a loss, board NMs, as well as wheel NMs. There are no opportunities for an LDW	This strategy is the same as 3.A and 3.B, but the lucky numbers are odd and even
4	(*10*) No. 6, (*10*) No. 8 (*10*) No. 18, (*10*) No. 20, and (*10*) No. 32	14%	360	There are chances for several types of NMs, as well as the chance for losses, but there is no chance for an LDW	Five bets each to the same value, placed in opposing corners on the roulette wheel
5.A	(*5*) No. 2, (*5*) No. 9, (*5*) No. 13, (*5*) No. 14, (*5*) No. 20, (*5*) No. 22, (*5*) No. 29, (*5*) No. 31, (*5*) No. 33, and (*5*) No. 36	27%	180	There are several chances for different types of NMs and losses but no LDWs	Ten bets each to the same value, scattered randomly across the board
5.B	(*10*) No. 2, (*10*) No. 9, (*10*) No. 13, (*10*) No. 14, (*5*) No. 20, (*1*) No. 22, (*1*) No. 29, (*1*) No. 31, (*1*) No. 33, and (*1*) No. 36	27%	36–360	There are several chances for NMs and no LDWs	Ten bets unevenly with even bet magnitude, scattered across the board in the same pattern as 5.A
6	(5) No. 3, (10) No. 26, (20) No. 0, (10) No. 32, and (5) on No. 15	14%	180–720	There is the possibility for several NMs and losses but no LDWs	5 bets placed on numbers next to each other on the wheel with the middle number having the greatest value bet, decreasing in value as it moves away from the middle
7	(15) 1st 12, (5) No. 0, (5) No. 13, (5) No. 14, (5) No. 15, (5) No. 16, (5) No. 17, and (5) No. 18	51%	45–180	There are no losses, LDWs, or board NMs	This strategy covers the first 18 numbers and by extension almost every other space on the roulette wheel
8	(45) black and (5) No. 0	51%	90–180	There is no chance of a board NM or LDW	​

To highlight the potential variability in outcome proportions, provided in the online supplementary materials (for all online suppl. material, see https://doi.org/10.1159/ear/acrag004) are additional data on 100, 500, and 1,000 randomised trials, the outcomes across all trials highlight similar outcome percentages. Overall, 2,000 spins were used as the number of simulated trials to highlight the law of probability, in that over the 2,000 trials a robust percentage of outcomes will be found without the volatility of smaller trial numbers. No player in reality may play 2,000 games of roulette; however, these data give insights into the common outcomes based on the roulette table.

### Statistical Analysis

The percentages of frequency for each outcome over the 11 strategies were calculated. This was done twice, firstly for each number being landed on once giving the true outcome percentages without the effect of randomisation, secondly for the randomised 2,000 trials. The overall winnings of each strategy were assessed to investigate whether they may differ; a between-subjects ANOVA was used. Partial eta squared is provided for the effect size using Cohen’s [18] guidelines (<0.01 is minimal to no effect, <0.06 is a small effect, <0.14 is a medium effect, >0.14 is a large effect).

## Results

### Outcome Types and Frequency

The proportionality of the outcome types was examined in two capacities. The first was based on an equal proportion of outcomes, with each number being the winning number only once (see Table 2), as well as the percentages based on the 2,000 games played with that strategy (see Table 3). The number of losses varied across the betting strategies; one strategy (strategy 7) had no loses, only NMs, while the other 10 strategies had a range of 8.2%–50.90% of loses. Two of the strategies could not produce any NMs, the range of probabilities for an NM across the strategies was 3.20%–63.84%. Nine of the strategies had no chance of developing an LDW; the chance of an LDW was between 10.20% and 15.25%. Finally, all strategies had a chance of getting a win; this ranged from 14.05% to 63.75%. Fourteen possible outcomes were initially identified across all of the strategies employed, but only 11 were encountered in the simulated data using these strategies. These data show that unlike many other casino games, roulette does not have a clear proportion of outcomes; instead, they can vary widely as a function of betting strategy. This furthers the concern that experiments using roulette as a game cannot have a preset sequence.

**Table 2.** Outcome proportions for each strategy with even distribution of wins

​	Loss	LDW	Win	Total NMs	1-Board NM	2-Board NMs	3-Board NMs	1-Wheel NM	2-Wheel NMs	1-Wheel 1-board NM	2-Wheel 1-board NM
Strategy 1	12 (32.43%)	6 (16.22%)	19 (51.35%)	N/A	N/A	N/A	N/A	N/A	N/A	N/A	N/A
Strategy 2	9 (24.32%)	4 (10.81%)	24 (64.87%)	N/A	N/A	N/A	N/A	N/A	N/A	N/A	N/A
Strategy 3.A	10 (27.03%)	N/A	18 (48.65%)	9 (24.32%)	7 (18.92%)	N/A	N/A	1 (2.70%)	N/A	1 (2.70%)	N/A
Strategy 3.B	15 (40.54%)	N/A	20 (54.05%)	2 (5.41%)	N/A	N/A	N/A	2 (5.41%)	N/A	N/A	N/A
Strategy 3.C	14 (37.84%)	N/A	19 (51.35%)	4 (10.81%)	3 (8.11%)	N/A	N/A	N/A	1 (2.70%)	N/A	N/A
Strategy 4	11 (29.73%)	N/A	5 (13.51%)	21 (56.76%)	7 (18.92%)	4 (10.81%)	N/A	7 (18.92%)	N/A	3 (8.11%)	N/A
Strategy 5.A	3 (8.11%)	N/A	10 (27.03%)	24 (64.86%)	11 (29.73%)	4 (10.81%)	1 (2.70%)	2 (5.41%)	1 (2.70%)	4 (10.81%)	1 (2.70%)
Strategy 5.B	3 (8.11%)	N/A	10 (27.03%)	24 (64.86%)	11 (29.73%)	4 (10.81%)	1 (2.70%)	2 (5.41%)	1 (2.70%)	4 (10.81%)	1 (2.70%)
Strategy 6	19 (51.35%)	N/A	5 (13.51%)	13 (35.13%)	10 (27.03%)	1 (2.70%)	N/A	1 (2.70%)	N/A	1 (2.70%)	N/A
Strategy 7	N/A	N/A	19 (51.35%)	18 (48.65%)	N/A	N/A	N/A	N/A	15 (40.54%)	N/A	3 (8.11%)
Strategy 8	17 (45.95%)	N/A	19 (51.35%)	1 (2.7)	N/A	N/A	N/A	1 (2.70%)	N/A	N/A	N/A

The current data represent the outcome proportions for each strategy across the 37 numbers on a roulette wheel.

NM, near miss; LDW, loss disguised as a win.

**Table 3.** Outcome proportions for each strategy over 2,000 randomly generated trials

​	Loss	LDW	Win	Total NMs	1-Board NM	2-Board NMs	3-Board NMs	1-Wheel NM	2-Wheel NMs	1-Wheel 1-board NM	2-Wheel 1-board NM
Strategy 1	629 (31.45%)	305 (15.25%)	1,066 (53.30%)	N/A	N/A	N/A	N/A	N/A	N/A	N/A	N/A
Strategy 2	521 (26.05%)	204 (10.20%)	1,275 (63.75%)	N/A	N/A	N/A	N/A	N/A	N/A	N/A	N/A
Strategy 3.A	556 (27.80%)	N/A	1,002 (50.10%)	442 (22.10%)	341 (17.05%)	N/A	N/A	61 (3.05%)	N/A	40 (2.00%)	N/A
Strategy 3.B	826 (41.30%)	N/A	1,069 (53.45%)	105 (5.25%)	N/A	N/A	N/A	105 (5.25%)	N/A	N/A	N/A
Strategy 3.C	740 (37.00%)	N/A	1,036 (51.80%)	224 (11.20%)	170 (8.50%)	N/A	N/A	N/A	54 (2.70%)	N/A	N/A
Strategy 4	579 (28.95%)	N/A	285 (14.25%)	1,136 (56.80%)	366 (18.30%)	225 (11.25%)	N/A	376 (18.80%)	N/A	169 (8.45%)	N/A
Strategy 5.A	164 (8.20%)	N/A	559 (27.95%)	1,277 (63.84%)	598 (29.90%)	195 (9.45%)	55 (2.75%)	124 (6.20%)	49 (2.45%)	204 (10.20%)	52 (2.60%)
Strategy 5.B	183 (9.15%)	N/A	551 (27.55%)	1,266 (63.3%)	589 (29.45%)	189 (9.45%)	64 (3.20%)	109 (5.45%)	55 (2.75%)	207 (10.35%)	53 (2.65%)
Strategy 6	1,018 (50.90%)	N/A	281 (14.05%)	701 (35.05%)	544 (27.20%)	50 (2.50%)	N/A	54 (2.70%)	N/A	53 (2.65%)	N/A
Strategy 7	N/A	N/A	1,039 (51.95%)	961 (48.05%)	N/A	N/A	N/A	N/A	811 (40.55%)	N/A	150 (7.50%)
Strategy 8	917 (45.85%)	N/A	1,019 (50.95%)	64 (3.20%)	N/A	N/A	N/A	64 (3.20%)	N/A	N/A	N/A

The current data represent the outcome proportions for each strategy.

NM, near miss; LDW, loss disguised as a win.

### Strategy Winnings

The total winning of each of the strategies were examined. None of the strategies earned a final value that exceeded more or less than 5% of the initial pool of money 100,000 (see Table 4). A between-subjects ANOVA found that there were no significant differences between each of the strategies and the mean amount won (*F*[10, 21,989] = 0.37, *p* = 0.96, $\eta_{\eta }^{2}$ = 0.0002). Overall, this shows that none of the strategies employed consistently over the length of play resulted in more winning in the roulette game. Simply put, most strategies remade the credits that had been started with or close to.

**Table 4.** Amount of credits earned from each strategy

​	Total amount earned	Mean amount won (SD)
Strategy 1	100,052	50.03 (39.39)
Strategy 2	95,910	47.96 (32.93)
Strategy 3.A	98,400	49.20 (79.02)
Strategy 3.B	96,580	48.29 (66.27)
Strategy 3.C	95,930	47.97 (70.19)
Strategy 4	102,600	51.30 (125.87)
Strategy 5.A	100,620	50.31 (80.80)
Strategy 5.B	99,180	49.59 (114.04)
Strategy 6	101,340	50.67 (145.74)
Strategy 7	98,730	49.37 (66.82)
Strategy 8	95,940	47.97 (48.97)

## Discussion

The current study sought to understand the impact of differing roulette strategies on outcome distribution and overall winnings and over 22,000 roulette spins across eleven different strategies. Analysis indicates that different strategies did not significantly impact the total earned but did have a significant impact on outcome distribution. This has significant implications for designing an experimental roulette task.

Experimental research examining roulette gambling must balance having a pre-determined outcome sequence to control for outcome impact, and ecological validity through unconstrained betting. To this extent, we offer three suggestions to how research can balance these factors attributing their strengths and weaknesses. (1) To have a pre-determined outcome sequence, the research must artificially constrain the method of betting. This includes having a set number of bets (>2) that have to be placed each round at differing values (e.g., 1, 2, 5, and 10) and restricting the number of ½ and 1/3 odds bets that can be placed. This will allow for the prevalence of LDW’s and NMs to be controlled, conforming to the planned outcomes [3, 4, 19]. This allows for the examination of common variables of interest such as persistence and the effect of outcomes on arousal. (2) To use an ecologically valid betting method, a pseudo-randomised outcome sequence can be used, which using a logic-based system can determine the outcome based on what has been bet and the proportion of outcomes that have already been experienced. This will require a large number of trials to get a usable number of outcomes for statistical analysis but would allow for the immediate assessment of the psychological impact of outcomes. However, as the total sequence is randomised between participants there is little generalisability overall. There is still the potential that gamblers may use strategies that have no chance of NMs or LDWs. (3) Similar to option 2, this would use a true random outcome sequence and no betting restrictions. This can be preferred to option 2 where computing power cannot run the logic to alter outcomes. This option has the lowest generalisability as each gamblers experience is unique, and outcome prevalence distribution variability could become a confounding variable. Statistical modelling would have to incorporate the outcomes frequencies into any analysis to understand the effect of the outcome on the psychological variable of interest.

Research in other gambling games has led to changes in law, through the use of experimental rigour and controlled methodologies [5, 6]. Prominently, this is having a rigged outcome sequence to control the experiment and provide generalisability. Methods for researching roulette need this level of detail; otherwise, the evaluative work on the harms of harmful roulette gambling and alterations to the mechanics of the game cannot be completed.

### Outcomes

The frequency of the outcomes varied widely across each of the strategies. In real gambling, the variation in outcomes could result in a mixed level of persistence in roulette as there is no defined ratio of wins, LDWs, or NMs [20]. This additionally means that we can make no suggestions as to the common proportions of outcomes for experimental research to use as a fixed outcome sequence. Thus, any research replicating roulette will have to dictate their own variations of frequency in their outcomes sequence. This additionally means that an extinction outcome sequence will include the opportunity for LDWs and NMs unless the betting methods are artificially constrained. The number of LDWs was lower than initially presumed with most of the tested strategies having no potential for LDWs; instead, there were more types of NMs.

The results have indicated that in roulette a gambler can experience any of the outcomes at the same time. In that, the gambler’s strategy on a given spin may result in an LDW, an NM, and a true loss all at once. Similarly, they may experience several wins at different magnitudes at once. However, the psychological impact of experiencing all of these outcomes in the same spin is not yet understood and whether they will override each other. Future research needs to explore the psychological impact and whether this impacts future gambling behaviours (e.g., persistence or bet size). In person, roulette can be a multiplayer game; there is the potential of other players outcomes influencing the perception of their own outcome. Gunnarsson et al. [21] found that in blackjack, the presence of another player experiencing an NM increased the participants perceived closeness to win. A crowded roulette table with several players may influence the individual’s perception of their own NM based on the other players outcomes.

Each of the strategies showed a large variation in the number of outcomes, with the wins varying from 14.05% to 63.75%. Despite this large variation in win potential and magnitudes of wins, there was no difference in the number of credits won by any of the strategies, meaning there was no difference in volatility. The strategies with higher win frequencies typically had lower win magnitudes and vice versa. Over the 2,000 random trials in the games, all of the strategies were within 5,000 credits of the initial 100,000. This shows that none of the strategies used in this study – which varied widely – are significantly more or less beneficial to the gambler. It can therefore be argued that any platform claiming to have a “winning” strategy is in fact highly unlikely.

### Near Misses

In regard to the types of NMs, for a number of strategies (4, 5.A, and 5.B), the prevalence of NMs increased to above 50% of spins. This is above the 30% of spins identified in previous research as optimally driving persistence [8, 22]. Similarly, some other strategies (3.B, 3.C, and 8) had NMs below 30%, which would have a similar effect in reducing persistence of play. NMs in roulette are more complex than many other games, given the number of types that have been found in this research. The psychological effect of the multiple NM types may have a differing effect than a singular NM. As the current data are theoretical, we cannot argue whether this will increase or decrease the effect on persistence. This effect may however be nullified as the number of NMs compounds, to where a quadruple-board double-wheel NM may cause the player to believe the game is against them. This additional effect to the NM may also be moderated by the strategy employed and whether this increases or decreases the NM frequency.

The types of NM outcomes warrants further research. In this study, an NM only refers to the direct neighbour of a bet space on the wheel or board. Based on Dixon’s [3] findings that the closer the bet to the winning number the greater the perceived closeness to a win, it may not only be direct neighbours that result in an NM. For the wheel NM, there is the potential for an *n* + 1 effect where the magnitude of the NM diminishes for every additional space between the bet and where the ball lands. For board NMs, the present research only considered the role of the direct horizontal and vertical neighbours on the board as NMs. Therefore, there is the possibility that the diagonal neighbours may also have an NM effect. Further, there is also the possibility that the next logical number is in the following column and thus not next to each other (i.e., 3 and 4, see Fig. 1), this may also result in an NM as it is the next number. Each of these issues may individually alter the impact of an NM on the player.

A further key issue is the consideration that in casinos a roulette board is large and distinct from the wheel. Eye tracking could be used to identify the difference between wheel NMs and board NMs as some participants may not notice an NM as they were not looking at the correct section. Online roulette games, particularly mobile games, do not show both the wheel and board at the same time due to the size of the screen, which may also limit the perception of the NM. This may impact gamblers persistence without conscious recognition.

## Conclusions

Simulated data have shown that the outcomes for a roulette wheel are more complex that initially presumed. Outcome frequencies vary based on the strategy employed, and overall, none of the strategies resulted in more money being earned over the 2,000 trials. Future research examining roulette gambling must strike a balance between experimental control and ecology validity by artificially restraining betting options, thus reducing the ecological validity, increasing the experimental control, or upholding the ecological validity and decreasing experimental control. Research in other forms of gambling has effectively informed policy and practice to reduce the damages of gambling. The ability to experimentally manipulate and examine gambling behaviours in roulette can allow for the same kind of research as other areas of gambling research to be conducted and create the opportunities for researchers to inform successful harm reduction and minimisation strategies.

## Statement of Ethics

An ethics statement was not required for this study type since no human or animal subjects or materials were used.

## Conflict of Interest Statement

S.S. has received funding for research from UKRI, NIHR, SSA, and Greo, is a member of the Executive Committee of the Academic Forum for the Study of Gambling, which is funded by Greo, via regulatory settlement funding from the Gambling Commission, and has also received travel expenses from the Current Advances in Gambling Research conference and the Alberta Gaming Research Institute. M.K.P. was employed by S.S. for research funded by the UKRI.

## Funding Sources

This research was funded by a UKRI Future Leader’s Fellowship, awarded to S.S. The funder had no role in the design, data collection, data analysis, and reporting of this study.

## Author Contributions

M.K.P. developed the simulation and generated the data used in the present research. Both S.S. and M.K.P. contributed towards the design of the project and the write-up of the manuscript and its revisions.

## Data Availability

The data that support the findings of this study can be found via the following link https://osf.io/7fvz9/overview?view_only=d1737b82e83e4615a1aa2caa9f332e12.
